# Infarction of middle third posterior cortex of kidney: a complication of extended pyelolithotomy, intra-operative electrohydraulic lithotripsy and extraction of calyceal stones under vision using stone basket and flexible cystoscope in a spinal cord injury patient – a case report

**DOI:** 10.1186/1757-1626-2-93

**Published:** 2009-01-28

**Authors:** Subramanian Vaidyanathan, Peter L Hughes, Gurpreet Singh, Bakul M Soni

**Affiliations:** 1 Regional Spinal Injuries Centre, District General Hospital, Southport PR8 6PN, UK; 2 Department of Radiology, District General Hospital, Southport PR8 6PN, UK; 3 Department of Urology, District General Hospital, Southport PR8 6PN, UK

## Abstract

**Background:**

Spinal cord injury produces multiple systemic and metabolic alterations. A decrease in micro vascular blood flow to liver, spleen and muscle has been described following spinal cord injury.

**Case presentation:**

We present a 46-year-old male patient with C-4 complete tetraplegia, who developed a large stag horn calculus with branches in upper, middle and lower calyces of left kidney. This patient underwent Gil-Vernet extended pyelolithotomy and required intra-operative electrohydraulic lithotripsy and retrieval of stones from upper, middle and lower calyces using flexible cystoscope and stone basket. Computed tomography, performed eighteen days after surgery, showed multiple areas of non-enhancing cortex posteriorly and in the upper pole, suggestive of focal infarction. Magnetic resonance imaging of left kidney confirmed the presence of an area of infarction in middle third of posterior cortex, but there was no evidence of trauma to posterior division of renal artery. Therefore, we postulate that compression of renal parenchyma by Gil-Vernet retractors during surgery, and firm pressure that was applied over the middle of kidney for prolonged periods while several attempts were being made to retrieve fragments of calculi from renal calyces, led to ischaemia and subsequently, infarction of mid-third posterior cortex of left kidney.

**Conclusion:**

This case illustrates importance of gentle handling of kidney during extended pyelolithotomy in order to prevent subtle renal trauma, which may be detected only by advanced imaging studies. Further, spinal cord physicians should take a pragmatic approach to management of stones located inside renal calyces. Both spinal cord injury patients and their physicians should remember that in our enthusiasm to achieve complete clearance of stones embedded deeply within renal calyces, we could produce irreversible injury to kidney, as indeed happened in this patient. *Therefore, emphasis should be placed on prevention of struvite renal calculi by discarding indwelling urinary catheters and eliminating Proteus bacteriuria*.

## Background

In neurosurgery, a microscope is used to allow the surgeon to work on intra cranial structures, which are located deeply in the brain. During such an operative procedure, considerable retraction of brain may be required for adequate exposure. Although retraction of brain aids in exposure, such retraction can cause secondary brain damage. Damage to brain occurs not only due to direct effect of the retractor on the cortical surface, but also because of the pressure, which is generated under the retractor on the brain tissue. Pressure applied to brain by these two mechanisms lead to a compromise in local cerebral blood flow and local cerebral perfusion pressure, thus causing cerebral ischaemia [1]. This neurosurgical scenario can well be applied to situations when open surgical procedures are carried out for retrieval of calculi embedded in renal calyces. For example, extended pyelolithotomy may be a straightforward procedure when main bulk of stone is located within renal pelvis, but the need for retraction of renal parenchyma is increased if stones are located deeply inside the calyces. The risk of generating ischaemic conditions is enhanced when the kidney is tense. Further, the degree of renal ischaemia is aggravated by associated systemic conditions such as hypotension, hypoxaemia, and hypercapnia.

We present a 46-year-old male patient with C-4 complete tetraplegia, who developed infarction of middle third posterior cortex of kidney. This patient had a large stag horn calculus in left kidney with branches in upper, middle and lower calyces. In our spinal unit, we prefer open surgery for removal of large stag horn calculus from kidney and therefore, this patient underwent Gil-Vernet extended pyelolithotomy and required intra-operative electrohydraulic lithotripsy and retrieval of stones from upper, middle and lower calyces using flexible cystoscope and stone basket. The role of open surgery for large stag horn calculus versus percutaneous nephrolithotripsy is controversial. Probably we fall in the category of "rural" urologists rather than "metropolitan" urologists, as only 22% of urban and metropolitan urologists would offer anatrophic nephrolithotomy [2].

Computed tomography, performed eighteen days after surgery, showed multiple areas of non-enhancing cortex posteriorly and in the upper pole, suggestive of focal infarction. Magnetic resonance imaging of kidneys confirmed the presence of an area of infarction in middle third of posterior cortex, but there was no evidence of trauma to posterior division of renal artery. Therefore, we postulate that compression of renal parenchyma by Gil-Vernet retractors, and firm pressure that was applied over the middle of kidney for prolonged periods while several attempts were being made to retrieve fragments of calculi from renal calyces, led to ischaemia and subsequently, infarction of mid-third posterior cortex.

## Case presentation

A 40-year-old Caucasian man sustained C-4 complete tetraplegia after a gunshot wound to his neck while on holiday in Spain in March 2002. This patient sustained injuries to left carotid artery and jugular vein, which were surgically ligated. It was also noted that he had injury to the left side of C-5 vertebra with epidural haematoma and leakage of cerebrospinal fluid for which he underwent surgery. Tracheostomy was done and he was on a ventilator. He had recurrent partial collapse of right lung. He was successfully weaned off the ventilator.

This patient had indwelling urethral catheter for drainage of urinary bladder. In 2003, ultrasound of right kidney showed marked right hydronephrosis. The bladder outline appeared normal. MAG-3 renogram performed in August 2003, showed no noticeable function in right kidney. There was normal uptake and excretion by the left kidney. Relative function of left kidney was 100% whereas relative function of right kidney was 0%. The MAG-3 renogram confirmed that the right kidney was non-functioning. In June 2006, this patient developed catheter blockages frequently. Flexible cystoscopy showed several calculi in urinary bladder. X-ray of abdomen taken in June 2006, showed middle and lower pole calculi in left kidney. On 30 June 2006, cystoscopy and electrohydraulic lithotripsy of vesical calculi were carried out.

**Figure 1:**
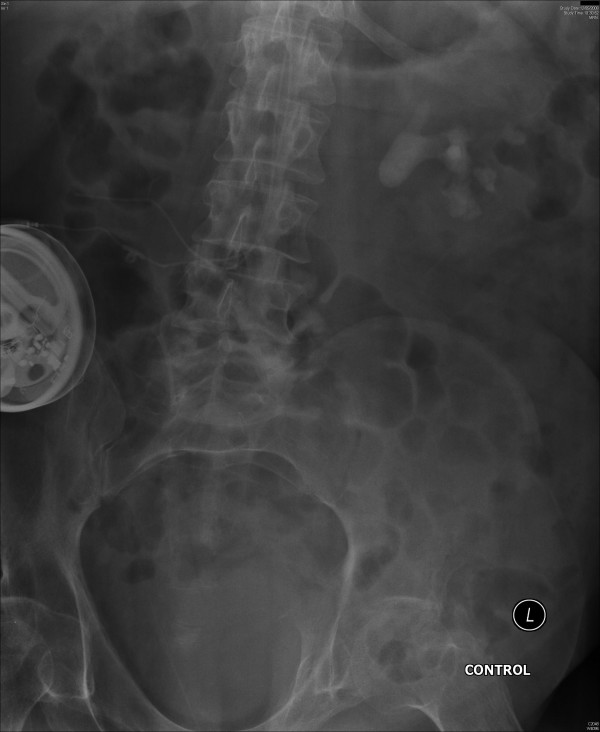
**X-ray of kidneys (12 May 2008) showed stag horn calculus in left kidney**.

**Figure 2:**
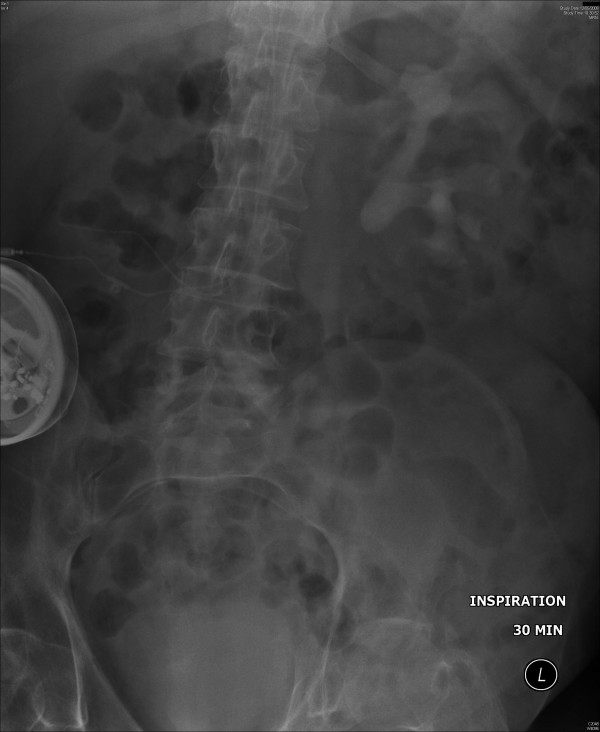
**Intravenous urography (12 May 2008), 30 minutes film showed moderate hydronephrosis**. No function was seen in right kidney.

On 10 April 2008, serum creatinine was 74 umol/L. Creatinine clearance was 79 mL/minute. In May 2008, this patient developed sweating and headache. There was blood in the urine bag. Intravenous urography showed stag horn calculus in left kidney (Figure 1) and moderate hydronephrosis. (Figure 2) No function was seen in right kidney.

Microbiology of urine, which was sample taken on 04 July 2008, showed growth of Proteus mirabilis. Ultrasound examination of kidneys, performed on 08 July 2008, showed moderate to marked hydronephrosis on the right with marked cortical thinning. Urine within the collecting system looked clear. Left kidney measured 12 cm. There were at least three calculi in the mid and lower pole calyces. There was no hydronephrosis or perinephric collection. The bladder outline was normal; no bladder calculi.

**Figure 3:**
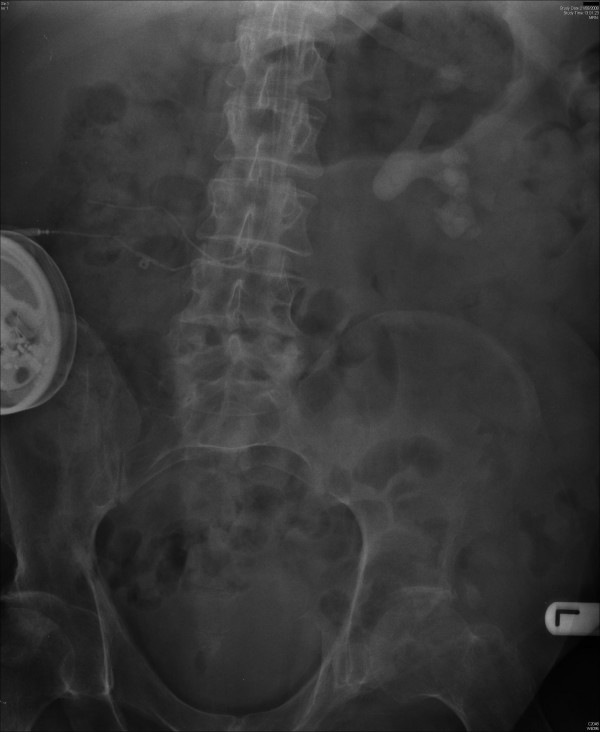
**X-ray of kidneys, which was taken on 21 August 2008, showed stag horn calculus with branches in upper, middle and lower calyces**.

The patient agreed to undergo open surgery for removal of stones from the left kidney. A sample of urine was sent for microbiology on 20 August 2008 so that the most appropriate antibiotic could be prescribed for surgery [3]. Urine culture yielded growth of Proteus species, Pseudomonas aeruginosa, and Enterococcus faecalis; all these organisms were sensitive to Tazocin. Tazocin 2.25 g contained piperacillin 2 g (as sodium salt) and tazobactam 250 mg (as sodium salt). X-ray of kidneys, which was taken on 21 August 2008, showed stag horn calculus with branches in upper, middle and lower calyces. (Figure 3) On 22 August 2008, left extended pyelolithotomy was carried out under inhalational anaesthesia. Anaesthesia was started at 0845 hours. His blood pressure was 124/76 mm hg. When the patient was turned to lateral position, there was a transient decrease in blood pressure to just less than 70/40 mm Hg. Subsequently, blood pressure was maintained around 100/70 mm Hg. Cardiovascular status and urine output remained stable during the whole operative procedure.

The twelfth rib was resected. Left kidney was mobilised. Left ureter was isolated. Fat around renal pelvis was freed. Gil-Vernet retractors (9 mm and 11 mm) were used to lift the renal parenchyma and thereby visualise neck of calyces. A horse-shoe-shaped incision was made over renal pelvis. A large stone was removed from renal pelvis. The renal calyces were flushed vigorously with 0.9% sodium chloride and small calculi were washed out. Flexible cystoscope was inserted through renal pelvis to inspect renal calyces. There were large calculi located within renal calyces. Electrohydraulic lithotripsy (EHL) of stones, which were located inside calyces, was carried out using flexible cystoscope and ureteric EHL probe. Stone fragments were removed under vision after inserting flexible cystoscope inside the calyces and using either stone basket or grasping forceps. During these procedures (saline washouts, electrohydraulic lithotripsy, and stone retrieval by basket), the kidney was stabilised and held firmly between thumb and fingers. There was not excessive bleeding at any time. After removing as many stones as possible from the calyces, a JJ stent was inserted in the ureter. Pelvis was closed and peri-renal fat was approximated over the kidney. Surgery finished around 1500 hours. No contrast was administered during surgery. The intra-surgery volume balance remained appropriate throughout surgery.

Following surgery, serum creatinine concentration increased from 91 umol/L to 220 umol/L (Table 1). On 29 August 2008, serum creatinine was 184 umol/L. Creatinine clearance had decreased to 31 mL/minute. Lactic dehydrogenase (LDH) levels in blood were not estimated. On thirteenth post-operative day (04 September 2008), there was large amount of bleeding through drainage site.

**Table 1:** Serum creatinine concentration before and after extended pyelolithotomy, which was performed on 22 August 2008

Date	Serum creatinine (umol/L)
20 August 2008	91

23 August 2008	220

24 August 2008	229

26 August 2008	207

27 August 2008	191

29 August 2008	184

10 September 2008	191

14 September 2008	231

24 September 2008	181

04 October 2008	177

18 October 2008	173

18 November 2008	208

21 November 2008	236

22 November 2008	232

**Figure 4:**
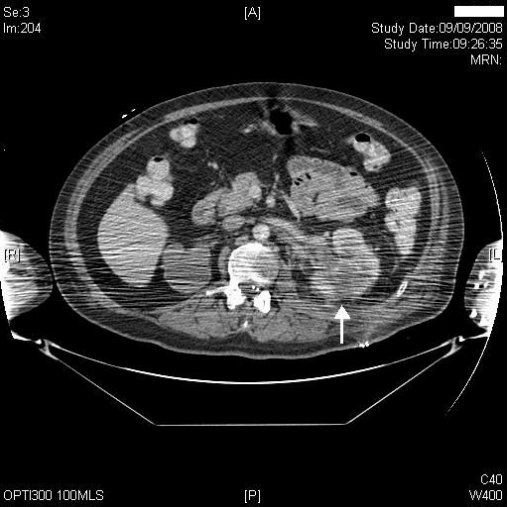
**CT of kidneys, performed on 09 September 2008 (arterial phase) showed an area of non-enhancement in posterior cortex (middle third) of left kidney (arrow)**.

**Figure 5:**
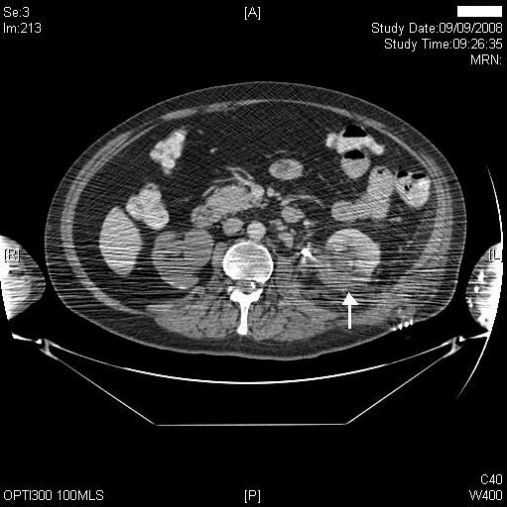
**CT of kidneys, performed on 09 September 2008 (arterial phase), showed non-perfusion in posterior cortex of left kidney (arrow); left ureteric stent in place; marked hydronephrosis of right kidney**.

Therefore, on 09 September 2008, plain and arterial phase scans through the kidneys followed by portal venous scan of the entire abdomen and pelvis was performed. There were extensive artefacts from the patient's arms, which he could not place above his head. This decreased image quality. The plain scan showed four residual calculi in upper, mid and lower poles of the left kidney. The arterial phase showed no large pseudo aneurysms, but due to the artefacts, smaller aneurysms could not be ruled out. There was no large old haematoma. In the portal venous phase of the scan, there were multiple areas of non-enhancing cortex posteriorly and in the upper pole of left kidney (Figures 4 and 5), suggestive of focal infarction. Mildly enlarged regional lymph nodes were seen, which was likely to be reactive following the previous operation. The proximal curl of the double J stent was lying in the proximal ureter. There were inflammatory changes in the peri-renal fat. The right kidney was grossly hydronephrotic with very little residual cortex, as was previously seen on ultrasound.

**Figure 6:**
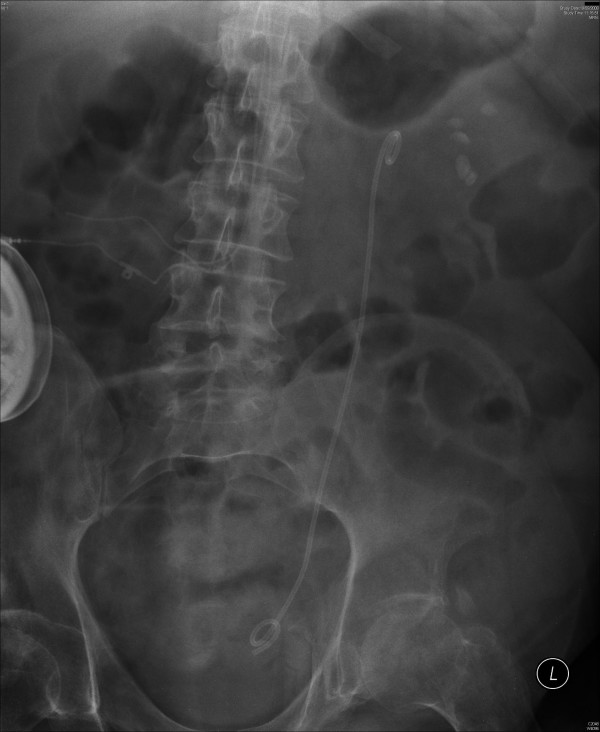
**X-ray of abdomen, taken on 19 September 2008, showed significant reduction in the bulk of the previously noted stag horn renal calculus; small residual calculi present in middle and lower pole calyces**.

X-ray of abdomen, taken on 19 September 2008, showed significant reduction in the bulk of the previously noted renal calculus disease. (Figure 6) The stag horn component has been removed. Some calyceal calculi in the mid and lower poles persist. There were two calculi in lower calyx and their combined length was 17 mm. No ureteric or bladder calculi were seen. On 16 October 2008, extracorporeal shock wave lithotripsy was performed. A total of 5000 shock waves were delivered to several stones in left kidney. This patient did not require analgesia or sedation for shock wave lithotripsy.

**Figure 7:**
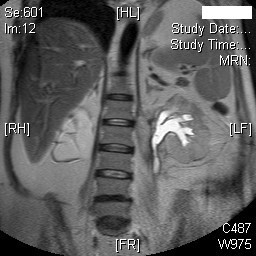
**MR Urography (18 November 2008) showed mild to moderate left hydronephrosis; cortical thinning of right kidney with marked hydronephrosis**.

**Figure 8:**
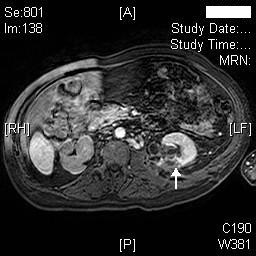
**Axial MR of kidneys (Arterial phase) performed on 18 November 2008, shows area of non-perfusion in posterior cortex, mid-pole of left kidney (arrow)**.

**Figure 9:**
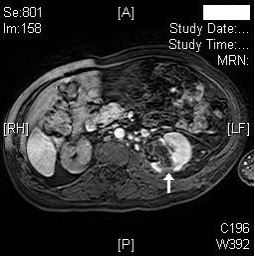
**Another section of the axial MR of kidneys (Arterial phase) performed on 18 November 2008 shows non-perfusion in posterior cortex, mid-pole of left kidney (arrow)**.

Dynamic magnetic resonance imaging (MRI) of kidneys was performed on 18 November 2008. MRI showed marked right hydronephrosis with marked cortical thinning of right kidney. Mild to moderate left hydronephrosis was present. (Figure 7) Calculi were seen in left kidney. There was some thinning of the posterior cortex of the left kidney and on the post contrast dynamic scans, there was an area measuring 2 cm × 3 cm that was not perfused in the middle third posterior cortex consistent with infarction at this site (Figures 8 and 9); no other abnormality was noted. Mild splenomegaly was seen.

The biochemical composition of stones removed from left kidney was: calcium phosphate – 48%; magnesium ammonium phosphate – 52%. This patient is scheduled to undergo another session of shock wave lithotripsy to fragment residual stones in lower and middle calyces.

In order to prevent further deterioration in renal function, this patient was advised to avoid drugs such as non-steroidal anti-inflammatory agents, which might cause renal damage. We explained to the patient and his family members that a small area in the back of left kidney has undergone infarction and therefore, carers should not apply excessive force or pressure on left loin while hoisting, transferring, and turning this patient. This patient was advised to discard indwelling urinary catheter so that the chances of urine infections and in particular, Proteus bacteriuria, might be decreased. He was prescribed modified-release oxybutynin 10 mg once daily and his family members would perform intermittent catheterisations 4 to 5 times a day.

## Discussion

Injury to posterior division of renal artery has been described following pyelolithotomy [4]. But in our patient, computed tomography of kidneys, which was performed 18 days after surgery, showed no abnormality of renal artery or its major branches. However, CT scan revealed multiple areas of non-enhancing cortex posteriorly and in the upper pole, suggestive of focal infarction. Differential diagnosis would be focal renal infection. But this patient did not show any clinical symptom of infection such as temperature, sweating, increased spasms, or feeling unwell. The surgical wound looked healthy. There was no discharge of pus either from the surgical incision or from drainage tube site. Therefore, we discounted the possibility of focal renal infection. In the absence of any injury to renal artery or its branches, we postulate that infarction of posterior cortex of kidney was probably due to ischaemia caused by local trauma.

Kitagawa and Iriyama [5] reported hepatic infarction in four patients, who underwent surgery for gastric cancer. Hepatic infarction occurred in two of these four patients due to persistent pressure on the folded liver by a retractor during surgery. The causative factor for hepatic infarction was pressure applied by retractor, was confirmed by post-operative computed tomography scanning, which revealed infarction despite preservation of hepatic artery.

Spinal cord injury produces multiple systemic and metabolic alterations. Spinal cord injury was associated with significant decreases in microvascular blood flow in liver, spleen, muscle and fore footpad skin [6]. Tetraplegic subjects demonstrate instability of cutaneous microvascular blood flow, which is related to the severity of paralysis [7]. Long-term follow-up of renal function after spinal cord injury revealed an over-all mean decrease in effective renal plasma flow of 4.5 ml. per year. Factors associated with a statistically significant reduction in effective renal plasma flow included age, gender, renal calculi, quadriplegia, and a history of chills and fever [8]. Our patient had tetraplegia, renal calculi, and developed urine infections in the past; all these factors contribute to reduced renal function. It is possible that micro vascular blood flow to parts of kidney got compromised when pressure was applied on renal cortex by Gil-Vernet retractors, which were used to lift renal parenchyma, and by subsequent operative procedures to remove stones embedded within calyces. Had we not performed CT and MRI of kidneys after surgery, we would not have detected focal infarction of kidney. We chose to perform dynamic magnetic resonance imaging of left kidney to assess perfusion of renal parenchyma because of the combined value of anatomical and functional information provided, as well as of specific contrast patterns that can be observed non-invasively [9,10].

During follow-up of spinal cord injury patients who undergo extended pyelolithotomy, only plain X-ray of kidneys is taken usually to assess presence of residual calculi; CT or MRI scans are not performed routinely. It is not possible to diagnose focal infarction of renal cortex by plain X-ray of kidneys; only advanced imaging studies will help to detect focal infarction of renal parenchyma. Therefore, we do not know whether infarction of middle third posterior cortex of kidney that was observed in this patient, is a unique occurrence or, happens not uncommonly but goes unnoticed.

This case illustrates importance of gentle handling of kidney during extended pyelolithotomy in order to prevent subtle renal trauma such as focal infarction, which can be detected only by advanced imaging studies. Further, spinal cord physicians should take a pragmatic approach to the problem of complete clearance of stones from renal calyces. Only18 (62%) of 29 patients with spinal neuropathy, who underwent percutaneous nephrolithotomy between October 1995 and January 2002, were rendered free of renal calculi after the initial percutaneous nephrolithotomy [11]. In Austin and Repatriation Medical Centre, Melbourne, Australia, between 1982 and 1996, 58 patients (3.5% of the spinal cord injury population) were treated for a total of 144 episodes of struvite calculi. Although clearance of stones was achieved in 87% of patients, *recurrent stones occurred in 69% of patients *[12]. Proteus bacteriuria has been shown to be significantly associated with urinary stones [13]. Similarly, the group of spinal cord injury patients developing struvite stones had a significantly higher incidence (49%) of indwelling catheters [12]. Our patient had Proteus bacteriuria and indwelling urethral catheter drainage, which are risk factors for recurrence of stone urolithiasis.

Spinal cord injury patients and their physicians should remember that in our enthusiasm to achieve complete clearance of stones from calyces, we might produce irreversible trauma to kidney, as indeed happened in this patient. *Therefore, emphasis should be placed on prevention of struvite renal calculi by discarding indwelling urinary catheters and eliminating Proteus bacteriuria*. But this requires motivation of spinal cord injury patient, carers and health professionals. Even today, organising intermittent catheterisations for a tetraplegic patient in the community remains a Herculean task [14].

## Consent

Written informed consent was obtained from the patient for publication of this case report and accompanying images. A copy of the written consent is available for review by the Editor-in-Chief of this journal.

## Competing interests

The authors declare that they have no competing interests.

## Authors' contributions

SV developed the concept and wrote the draft. PH reviewed CT and MRI images. GS and SV performed the surgical procedure. All authors contributed to patient care.
